# COVID-19 Pandemic: Stress, Anxiety, and Depression Levels Highest amongst Indigenous Peoples in Alberta

**DOI:** 10.3390/bs11090115

**Published:** 2021-08-24

**Authors:** Mobolaji A. Lawal, Reham Shalaby, Chidi Chima, Wesley Vuong, Marianne Hrabok, April Gusnowski, Shireen Surood, Andrew J. Greenshaw, Vincent I. O. Agyapong

**Affiliations:** 1 Department of Psychiatry, Faculty of Medicine and Dentistry, University of Alberta, Edmonton, AB T6G 2B, Canada; mobolaji@ualberta.ca (M.A.L.); rshalaby@ualberta.ca (R.S.); Chidi.Chima@albertahealthservices.ca (C.C.); marianne.hrabok1@ucalgary.ca (M.H.); andy.greenshaw@ualberta.ca (A.J.G.); 2 Addiction and Mental Health, Alberta Health Services, Edmonton, AB T5K 2J5, Canada; Wesley.Vuong@albertahealthservices.ca (W.V.); April.Gusnowski@albertahealthservices.ca (A.G.); shireen.surood@albertahealthservices.ca (S.S.); 3 Cumming School of Medicine, University of Calgary, Calgary, AB T2N 4Z6, Canada

**Keywords:** COVID-19, mobile phones, text, anxiety, depression, stress, psychological distress, pandemic, ethnic groups, Indigenous people

## Abstract

This study explores differences in stress, anxiety, and depression experienced by different ethnic groups during the COVID-19 pandemic. This was a cross-sectional online survey of subscribers of the COVID-19 Text4Hope text messaging program in Alberta. Stress, anxiety, and depression were measured among Caucasian, Indigenous, Asian, and other ethnic groups using the Perceived Stress Scale (PSS)-10, Generalized Anxiety Disorder (GAD)-7, and Patient Health Questionnaire (PHQ)-9 scales, respectively. The burden of depression and stress were significantly higher in Indigenous populations than in both Caucasian and Asian ethnic groups. The mean difference between Indigenous and Caucasian for PHQ-9 scores was 1.79, 95% CI of 0.74 to 2.84, *p* < 0.01 and for PSS-10 it was 1.92, 95% CI of 0.86 to 2.98, *p* < 0.01). The mean difference between Indigenous and Asian for PHQ-9 scores was 1.76, 95% CI of 0.34 to 3.19, *p* = 0.01 and for PSS-10 it was 2.02, 95% CI of 0.63 to 3.41, *p* < 0.01. However, Indigenous participant burden of anxiety was only significantly higher than Asian participants’ (mean difference for GAD-7 was 1.91, 95% CI of 0.65 to 3.18, *p* < 0.01). Indigenous people in Alberta have higher burden of mental illnesses during the COVID-19 pandemic. These findings are helpful for service planning and delivery.

## 1. Introduction

Several studies have reported increased or high levels of psychological distress and perceived mental illnesses in different populations during a pandemic or epidemic of a disease [1,2,3,4,5,6,7,8,9,10] and during mass disasters [11,12,13]. By early 2020, the Chinese Center for Disease Control and Prevention investigated clusters of patients with pneumonia of unknown etiology in Wuhan, leading to the isolation of a novel coronavirus (2019-nCoV) [14]. The World Health Organization (WHO) declared a global pandemic [15] on 11 March 2020 and by July, there were around 10 million coronavirus disease (COVID-19) cases and approximately 510,000 COVID-related deaths. Canada reported about 104,000 COVID cases with approximately 8600 related deaths in the same period [16].

The WHO has reported that one in four people will suffer mental or neurological disorders [17]. The 2017 Global Burden of Disease Study revealed that, depressive disorders and anxiety disorders were the third and eighth leading causes of years lived with disability (YLDs), respectively [18,19]. In Canada, mental health and substance use disorders are the second leading cause of YLDs. Around 20% of Canadian residents experience mental illness each year, most frequently mood and anxiety disorders [20]. Suicide can occur in psychotic and affective disorders but it is a core symptom of depressive disorder [21] and around 4000 deaths by suicide occur in Canada annually [22]. The total annual cost of mental illnesses to the Canadian economy was estimated at $51 billion (CAD) in 2008 with a projected increase to $2.5 trillion (CAD) by 2041 [20].

Indigenous peoples are a diverse set of communities living across Canada, comprising First Nations, Métis, and Inuit populations [23]. Indigenous peoples constituted 4.9% of the population of Canada in 2016 [24]. Collectively, Indigenous peoples carry the highest burden of physical and mental illnesses in Canada [25,26,27,28,29,30,31], a trend that extends to other countries with Indigenous populations [32,33,34,35,36,37]. However, one study reported similar prevalence of depression and anxiety for Indigenous and non-Indigenous populations in the Canadian province of British Columbia [38]. Additionally, a systematic review and meta-analysis study that combined studies of Indigenous people of the Americas reported significantly lower lifetime prevalent rates of generalized anxiety, panic, and all depressive disorders in Indigenous populations. However, post-traumatic stress disorder (PTSD) and social phobia were significantly higher relative to the non-Indigenous population [39]. Despite these findings, the overwhelming majority of studies [25,26,27,28,29,30,31,32,33,34,35,36] and a United Nations report [37] agreed that the burdens of physical and of mental illnesses are generally higher in Indigenous peoples compared to non-Indigenous populations.

Worldwide, suicide rates among Indigenous peoples are generally significantly higher than in non-Indigenous populations [40]. In a 2019 Canadian study, relative to non-Indigenous persons, the suicide rate among Inuit peoples was nine times higher; three times higher among First Nations peoples; and two times higher among Métis peoples [41]. However, suicide rates varied by First Nations Band, with a reported suicide rate of zero about 60% of bands. In addition, suicide rates were highest in people aged 15–24 years among First Nations males and Inuit males and females [41]. The reasons for the disproportionately higher burden of physical and mental illnesses among the Canadian Indigenous populations is attributable to social determinants of health (e.g., education, housing, socioeconomic status, access to services, etc.). Indigenous peoples often experience inequities that predispose them to experience poorer health outcomes compared to non-Indigenous populations [42,43,44]. The Truth and Reconciliation Commission of Canada report detailed intergenerational trauma inflicted upon Indigenous peoples from the imposition of the residential school system and the Indian Act in Canada over several decades [45].

Numerous studies, using standardized questionnaires, have reported high levels of psychological distress, anxiety, and depression due to the COVID-19 pandemic [1,2,3,4,5,6,7,8,9,10,46,47]. The prevalence of anxiety and depression, as measured by different standardized questionnaires, varied widely during the COVID-19 pandemic. For depression, the prevalence ranged from 14.6% to 53.8% during the COVID-19 pandemic [1,2,4,6,9,46], in excess of expected worldwide prevalence of 2% to 6% in 2017 [48]. Reported prevalence for anxiety during the COVID-19 pandemic range from 8.3% to 50.3% [1,2,4,6,8,9,10,46], also far higher expected worldwide prevalence of 2.5% to 7% in 2017 [48]. For stress during the COVID-19 pandemic, estimated prevalence ranges from 7.6% to 53.8% [2,5,8,9,47]. These worldwide prevalence for anxiety disorders and depression used for comparison were gleaned from a combination of survey-, medical- and epidemiological data, and meta-regression modelling in cases where raw data is missing [48]. Different studies reported variable epidemiological risk factors that are significantly associated with increased prevalence of anxiety, depression, and stress, measured by standardized questionnaires, during the COVID-19 pandemic in different populations [1,2,3,4,5,6,7,9,10,46].

Risk factors positively associated with depression in these studies included age 21–40 years [4,10], female gender [2,9], alcohol use [10], negative affect, detachment, an acquaintance infected with COVID-19, prior medical problems, stressful situations [2], specific physical health symptoms, poor self-rated health status [9], being close to the epicenter of the outbreak [10], being quarantined or affected by quarantine [3,46], financial burden due to massive quarantine [6], and spending more time exposed to COVID-19 related news [6]. Risk factors reported to be significantly associated with increased prevalence of anxiety included age 20–46 years [1,2,4,10], female gender [2,9], negative affect, detachment, stressful situations, prior medical problems [2], family members infected with COVID-19 [2,10], being infected with COVID-19 [10], specific physical health symptoms, poor self-rated health status and student status [9], quarantine or affected by quarantine [7,46], financial burden due to massive quarantine [6], perceived impact of COVID-19 [7], and time spent exposed to COVID-19 related news information [6,10].

Most factors that are positively associated with anxiety and depression during the COVID-19 pandemic may also bey associated with stress. These include age 20–46 years [1,2,10], female gender [2,5,9], negative affect, detachment, having an acquaintance infected with COVID-19 [2], higher educational level, migrant status, being close to the epicenter of a COVID-19 outbreak [5], quarantined or affected by quarantine [46], student status, specific physical health symptoms, and poor self-rated health status [9]. Despite the positive association of these factors with anxiety, depression, and stress during the COVID-19 pandemic some studies reported no such relationship for several of these factors. For instance, gender had no effect on anxiety and depression in two of these studies [1,7]. However, in a global report of anxiety disorder and depression estimates, females were reported to have higher prevalence of depression compared to males (4.1% versus 2.7%, respectively). In the same report, females had higher prevalence of anxiety disorders than males (4.7% versus 2.8%, respectively) [48]. In that report, median estimates for prevalence of depression and anxiety were higher in females for all the countries considered.

Given the generally greater burden of health among Indigenous peoples in Canada, in the absence of published detailed mental health studies addressing this inequity, we hypothesized that perceived stress, anxiety, and depression, measured by standardized instruments, would be higher for among Indigenous persons relative to other ethnic groups.

Text4Hope is a texting service that delivers once-daily supportive messages to support individuals’ mental health during the COVID-19 pandemic. Text messages were based on cognitive-behavioral therapy concepts and developed by mental health professionals (i.e., psychiatrists, psychologists, and mental health therapists). The program offers mental health support, aims to ameliorate pandemic-related negative thoughts and feelings, and help individuals to develop healthy coping and resiliency skills [49]. The program was modeled after the Text4Mood [50] and Text4Support [51] mental health texting services provided to people in Alberta, Canada. Ultimately, such services are evidence-based, clinically relevant, and cost-effective interventions applied as a therapeutic alliance to mental healthcare services for individuals with depressive and drug use disorders [52,53,54,55]. The aim of this program is to close the psychological treatment gap experienced in healthcare systems during the COVID-19 pandemic.

The aim of this study was to determine the burden of anxiety, depression, and stress in relation to ethnicity for Text4Hope subscribers who completed the baseline survey.

## 2. Materials and Methods

A cross-sectional survey was used to explore mean differences in perceived stress, anxiety, and depression symptom scores among the various ethnic groupings for Text4Hope subscribers who completed the baseline survey. This study explored each participant’s demographic characteristics (gender, age, level of education, employment status, relationship status, and housing status) and stratified them by their self-identified ethnicity (Caucasian, Indigenous, Asian, and Other). The mean scores, standard deviations, and standard errors from the Perceived Stress Scale (PSS)-10 [56], Generalized Anxiety Disorder (GAD)-7 [57], and Patient Health Questionnaire (PHQ)-9 [58] for self-reported symptoms of stress, anxiety, and depression, respectively, were stratified by ethnicity. The PSS is a 10-item validated self-report questionnaire (with an associated Cronbach’s alpha of >0.70) which is used to assess the level of stress in the previous month. Each item on the scale is scored between 0 (never) to 5 (very often). Higher scores on the scale indicate higher levels of stress. The GAD-7 is a 7-item validated questionnaire (associated with a Cronbach’s alpha of 0.92) which is used to assess the self-reported levels of anxiety in respondents in the two weeks prior to assessment. Each item on the scale is scored between 0 (not at all) to 4 (nearly everyday). Higher scores on the scale indicate higher levels of anxiety. The PHQ-9 is a 9-item validated instrument (associated with a Cronbach’s alpha of 0.89) which used to diagnose and measure the severity of depression in general medical and mental health settings. Each of the 9 questionnaire items is scored between 0 (not at all) to 3 (nearly every day). Higher scores on the scale indicate higher levels of depression.

The mean scores for stress, anxiety, and depression were compared among the ethnic groups and their differences were calculated using appropriate statistical methods to determine the mean differences and a 95% confidence interval in their PSS-10, GAD-7, and PHQ-9 scores. These mean differences and their 95% confidence interval depict the burden of anxiety, depression, and stress for each ethnic group relative to the other ethnic groups.

### 2.1. Recruitment

The study recruitment procedures and sample size estimations have been described earlier [59,60,61,62]. In summary, Text4Hope was launched by AHS on 23 March 2020. The program’s aim was to send daily supportive text messages to help support Albertans dealing with mental health concerns due to the COVID-19 pandemic. An online survey link was sent to self-subscribers and requested demographic information including gender, age, ethnicity, education, relationship status, employment status, type of employment, housing status, isolation status, and clinical characteristics of stress, anxiety, depression and obsessive-compulsive symptoms. Clinical characteristics were assessed using validated scales for self-reported symptoms, including the PSS-10, GAD-7, and PHQ-9. Participant consent was implied by submission of subscribers’ survey responses. Ethical approval for the research study was obtained through the University of Alberta Health Research Ethics Board (Pro00086163). Data was collected between 24 March 2020 and 4 May 2020.

### 2.2. Statistical Methods

Data analysis was undertaken using IBM Statistical Package for Social Sciences (SPSS) Statistics for Windows, version 26 (IBM Corp., Armonk, NY., USA). Demographic characteristics of respondents were summarized in absolute numbers and percentages, by ethnic category. A one-way analysis of variance (One-Way ANOVA) with two tailed significance (*p* < 0.05) was used to assess statistical differences between the ethnic groups for corresponding mean scores on the PSS-10, GAD-7, and PHQ-9. For variables that did not violate the assumptions of homogeneity of variance in the mean scores on the ANOVA test, we performed a Turkey’s post hoc test to determine if there were statistically significant differences in the mean scores of the various clinical measures between the different ethnic groups. For variables which violated the homogeneity of variance assumption, we determined if there were statistically significant differences for the mean scores for the various clinical measures between the different ethnic groupings using the Welch F test and a Games–Howell post-hoc test (as these tests do not require groups to exhibit homogeneity of error variance).

## 3. Results

### 3.1. Demographic Characteristics of Respondents

Of the 44,992 subscribers who joined Text4Hope in the first 6 weeks, 8267 responded to the online survey invitation, yielding a 19.4% response rate. Of the 8267 respondents, 6685 (81.0%) identified as Caucasian, 302 (3.7%) identified as Indigenous, 407 (4.9%) identified as Asian, 756 (9.2%) identified as belonging to “Other” ethnic groups, and 103 (1.2%) did not identify their ethnicity. The other demographic characteristics of the respondents are displayed in Table 1.

In Table 1, the majority of respondents identified as female (*n* = 7079, 86.9%), aged between 26 and 60 years (*n* = 6340, 79.3%), had post-secondary education (*n* = 6932, 85.1%), were employed (*n* = 5956, 73.1%), were married, cohabiting, or partnered (*n* = 5773, 70.9%), and owned their own home (*n* = 5243, 65.7%).

### 3.2. Association between Ethnic Categories and Prevalence of Perceived Stress, Likely GAD, and Likely MDD

The data displayed in Table 2 illustrate prevalence rates for clinically meaningful stress, anxiety, and depression. These data suggest the prevalence of moderate to high stress, likely Generalized Anxiety Disorder (GAD), and likely Major Depressive Disorder (MDD) were highest in respondents that identified as Indigenous.

The mean score for all respondents (*n* = 7589) on the PSS-10 was 20.79 (SD = 6.83). For the PHQ-9 scale, the mean score for all the respondents (*n* = 7082) was 9.43 (SD = 6.29) and for the GAD-7 scale, the mean score for all respondents (*n* = 6944) was 9.68 (SD = 5.87). Chi square analysis revealed significant association between the prevalence of likely stress and depression and the ethnicity of the participants, but not for the likely anxiety symptoms.

Table 3 presents the means and standard deviations for each of the PSS-10, PHQ-9, and GAD-7 scales by ethnicity groups.

Table 3 suggests that respondents that self-identified as Indigenous had the highest mean scores on the PSS-10, PHQ-9, and GAD-7 scales, while respondents that self-identified as Caucasian or Asian had fairly similar mean scores on the PSS-10 and PHQ-9 but not the GAD-7 scales.

Table 4 represents the results of One-Way ANOVA comparing sums of squares between and within ethic groups for the PSS-10, PHQ-9, and GAD-7 scales.

Table 4 suggests that there were statistically significant differences between and within ethnic groups for scores on the PSS-10 (F = 10.013, *p* < 0.001), the PHQ-9 (F = 8.98, *p* < 0.001), and the GAD-7 (F = 5.18, *p* < 0.001). The Levene Statistic test of homogeneity of variances suggested no violation of the assumption of equality of means for the PSS-10 and the GAD-7 (*p* > 0.05) and so we ran the Turkey’s post-hoc test to determine statistically significant differences in the mean scores between the different ethnic groups as presented in Table 5 But in contrast, the Levene Statistic test of homogeneity of variances indicated a violation of the assumption of homogeneity of error variance for the PHQ-9 scores (*p* < 0.05). Consequently, we ran a Welch F test and a Games-Howell post-hoc test to determine statistically significant differences between mean scores on the PHQ-9 between the different ethnic groups. The Welch F test was statistically significant which confirms that the differences between the groups in terms of their mean PHQ-9 scores are statistically significant. The results of the Games–Howell post-hoc test is as presented in Table 5.

Table 5 suggests that respondents who self-identified as Indigenous had significantly higher mean scores on the GAD-7 compared to respondents that self-identified as Asian (Mean Difference = 1.91, 95% CI = 0.65–3.18 and *p* < 0.01) but not respondents that self-identified as Caucasian (*p* > 0.05) or “Other” ethnic groups (*p* > 0.05). On the other hand, respondents that identified as Caucasian had significantly higher mean scores on the GAD-7 compared to respondents that identified as Asian (Mean Difference = 1.04, 95% CI = 0.16–1.92, *p* = 0.01) but not respondents that identified as “Other” ethnic groups (*p* > 0.05). However, respondents that identified as Asian had significantly lower mean scores on the GAD-7 compared to respondents that identified as “Other” (Mean Difference= −1.10, 95% CI= −2.16 to −0.04, *p* < 0.05). Table 5 also suggests that respondents that identified as Indigenous had higher mean scores on the PSS-10 than both respondents that identified as Caucasian (Mean Difference = 1.92, 95% CI = 0.86–2.98, *p* < 0.01) or Asian (Mean Difference = 2.02, 95% CI = 0.63–3.41, *p* < 0.01) but not respondents that identified as “Other” ethnic groups (*p* = 0.13). There were no statistically significant differences in the mean scores between respondents that identified as Caucasian or Asian for the mean PSS-10 scores (*p* > 0.05). However, respondents that identified as Caucasian had significantly lower mean scores on the PSS-10 compared to respondents that identified as “Other” ethnic groups (Mean Difference = −0.87, 95% CI = −1.58 to −0.15, *p* < 0.05).

Table 5 suggests that respondents that identified as Indigenous had significantly higher mean scores on the PHQ-9 compared to respondents that identified as Asian (Mean Difference =1.76, 95% CI = 0.34–3.19 and *p* < 0.05) or Caucasian (Mean Difference = 1.79, 95% CI = 0.74–2.84, *p* < 0.01), but not those who identified as “Other” ethnic groups (*p* > 0.05). On the other hand, respondents that identified as Caucasian had significantly lower mean scores on the PHQ-9 compared to respondents that identified as “Other” ethnic groups (Mean Difference = −0.74, 95% CI = −1.46–−0.01, *p* = 0.05).

## 4. Discussion

To our knowledge, this is the first study to examine how depression, anxiety, and stress symptoms vary among ethnic groups in Canada during the COVID-19 pandemic. The demographics of our study population suggest that the demographic profile of subscribers of the Text4Hope program and Text4Mood programs [50] are comparable. Our study found that respondents identifying as Indigenous reported higher levels of depression, anxiety, and stress than other ethnic groups in Alberta, Canada as measured by the PHQ-9, GAD-7, and PSS-10, respectively. Persons who identified as Caucasian or Asian reported similar levels of depression and stress, but respondents that identified as Caucasian reported more anxiety than respondents that identified as Asian. However, the significantly lower mean GAD-7 score reported by respondents that identified as Asian, compared to other ethnic groups, is not surprising given that in a global study of anxiety disorders, Asian people had lower prevalence of anxiety disorders (2% to 4%) compared to the rest of the world (4% to 6%), with the exception of some countries in South America, Eastern Europe, and Finland with similar prevalence of anxiety disorders [48].

Overall, the prevalence of distress was high, which is consistent with previous research. For example, significant stress was reported by over 90% of respondents that identified as Indigenous, and significant anxiety and depression were reported by over 50%. High rates of anxiety, depression, and stress found in this study are consistent with previous research showing high levels of population-level distress during the pandemic [1,2,4,6,8,9,10,46], with rates grossly elevated compared to pre-COVID-19 estimates [48]. For instance, the prevalence of significant anxiety symptoms in our study of 47.1%, was similar to the rates reported in different studies across the world during the COVID-19 pandemic [1,2,4,6,8,9,10,46], yet is much higher than an estimated global pre-COVID-19 rate in 2017 of 2.5% to 7% for anxiety [48].

The COVID-19 pandemic has likely accentuated pre-existing differences in the burden of mental health. For example, although a higher burden of mental health conditions among Indigenous peoples compared to non-Indigenous people in Canada was reported prior to the pandemic [29,30,31,63,64,65], as well as in other countries with Indigenous populations [35,36,40], it is likely that COVID-19 has placed additional stressors on this group. The reasons for the disproportionately higher burden of physical and mental illnesses among Indigenous peoples may include inequalities related to social determinants of health (e.g., education, housing, socioeconomic status, access to services, etc.), which can place Indigenous peoples at greater risk of poorer health outcomes compared to non-Indigenous populations [42,43,44]. This pattern of data likely stems from broad societal events; for example, in Canada, Indigenous people have experienced intergenerational trauma related to the Residential School System and the Government of Canada’s Indian Act over decades [45]. It has been documented that natural disasters and public health crises exaggerate pre-existing social inequities, and our findings are consistent with this.

Our study has several limitations, including a lack of baseline data on the stress, anxiety, and depression levels immediately prior to the COVID-19 pandemic and related restrictions, limiting direct pre- and post-COVID-19 comparisons. Second, the response rate in our study of 19.4% was low. However, our sample size was greater than the sample size of 3693 needed for prevalence rates estimates for stress, anxiety, and depression in our overall sample of 44,992 subscribers or the 4200-sample size needed for prevalence rate estimates in the entire Alberta population with a confidence interval of 99% and a 2% margin of error. Third, our study likely evidences selection bias given the rather low response rate; specifically, it is possible that non-respondents may differ in a systematic way compared to respondents. For example, they may be more (or less) affected by the pandemic or may have limitations in literacy or English fluency. Similarly, our study has some limited generalizability. The respondents that self-identified as Indigenous in our study represented 3.7% of our sample, lower than estimated proportion of the Indigenous population in Canada (4.9%) [24]. Furthermore, the vast majority of our respondents identified as female (86.9%), which is an overrepresentation in our study relative to Canadian population estimates of 50.3% [66]. This may likely increase the prevalence of perceived anxiety and depression in our study population given that females reportedly have higher prevalence of anxiety disorders and depression in all the countries considered in a global study [48]. Finally, although the ANOVA analysis allowed for comparison of the stress, anxiety, and depression levels between all the ethnic groups as a strength, it did not take into account potential confounding factors such as gender, age, relationship status, employment status, and education status, which is a limitation as ethnicity is likely to be one of several key factors upon which vulnerability to mental health effects of COVID-19 would be based. In addition, other social determinants of health, along with co-morbid physical health conditions, are known to play a significant role in increasing vulnerability in times of crisis [11]. Any interventions aimed at mitigating mental health effects of COVID-19 must therefore take of all these various factors into account.

## 5. Conclusions

The results of our study indicate that stress, anxiety, and depression levels are high in the general population during the COVID-19 pandemic, and are significantly associated with specific risk factors, such as Indigenous ethnicity. These results suggest a need for population-level mental health support, with enhanced psychological intervention for specific groups, such as Indigenous persons. Future studies should consider using a prospective study design in randomly selected participants from the general population with participants screened by trained clinicians for the presence of depression, anxiety, and stress using standardized objective assessment instruments. This will minimize recall bias and ensure that study findings are generalizable to the entire population.

Despite several limitations, this study provides useful data about the mental health ethnicity correlates of the COVID-19 pandemic. These findings are important for government and healthcare planning to determine the nature and quality of services required to address mental health challenges arising during this pandemic, as well as future pandemics that enforce public health measures. Specifically, planning for and implementing virtual care programs as well as supportive text messages is a relatively low-cost and easily scalable means of supporting individuals during public health crises [53,67,68,69,70], especially appropriate for supporting persons at additional risk of negative outcomes [52,54,55].

## Figures and Tables

**Table 1:** Demographic characteristics of respondents by self-reported ethnicity.

Variables	Caucasian *N* (%)	Indigenous *N* (%)	Asian *N* (%)	Other *N* (%)	Overall *N* (%)
**Gender**					
Male	751 (76.5)	32 (3.3)	93 (9.5)	105 (10.7)	981 (12.0)
Female	5884 (83.1)	263 (3.7)	312 (4.4)	620 (8.8)	7079 (86.9)
Other	50 (55.6)	7 (7.8)	2 (2.2)	31 (34.4)	90 (1.1)
**Age**					
≤25	646 (71.3)	47 (5.2)	88 (7.9)	125 (13.8)	906 (11.1)
26–40	2355 (80.4)	124 (4.2)	188 (6.4)	261 (8.9)	2928 (36.6)
41–60	2905 (85.1)	111 (3.3)	113 (3.3)	283 (8.3)	3412 (42.7)
>60	673 (89.5)	13 (1.7)	10 (1.3)	56 (7.4)	752 (9.4)
**Education**					
Less than High School Diploma	214 (65.2)	35 (10.7)	17 (5.2)	62 (18.9)	328 (4.0)
High School Diploma	621 (77.7)	50 (6.3)	21 (2.6)	106 (13.3)	798 (9.8)
Post-Secondary Education	5815 (83.9)	212 (3.1)	388 (5.2)	547 (7.9)	6932 (85.1)
Other Education	35 (38.9)	4 (4.4)	8 (8.9)	43 (47.8)	90 (1.1)
**Employment Status**					
Employed	4970 (83.4)	196 (3.3)	300 (5.0)	490 (8.2)	5956 (73.1)
Unemployed	744 (78.2)	66 (6.9)	34 (3.6)	107 (11.3)	951 (11.7)
Retired	498 (89.9)	8 (1.4)	6 (1.1)	42 (7.6)	554 (6.8)
Students	310 (68.4)	25 (5.5)	53 (11.7)	65 (14.3)	453 (5.6)
Other	161 (68.8)	7 (3.0)	12 (5.5)	54 (23.1)	234 (2.9)
**Relationship Status**					
Married/Cohabiting/Partnered	4820 (83.5)	178 (3.1)	276 (4.8)	499 (8.6)	5773 (70.9)
Separated/Divorced	533 (86.7)	25 (4.1)	14 (2.3)	43 (7.0)	615 (7.5)
Widowed	117 (88.6)	4 (3.0)	1 (0.8)	10 (7.6)	132 (1.6)
Single	1167 (76.0)	90 (5.9)	110 (7.2)	168 (10.9)	1535 (18.8)
Other	47 (50.5)	5 (5.4)	3 (3.2)	38 (40.9)	93 (1.1)
**Housing Status**					
Own Home	4532 (86.3)	123 (2.3)	197 (3.8)	401 (7.6)	5243 (65.7)
Living with Family	533 (67.6)	43 (5.5)	99 (12.6)	113 (14.3)	788 (9.8)
Renting	1466 (78.0)	123 (6.5)	93 (4.9)	197 (10.5)	1879 (23.5)
Other	39 (48.1)	6 (7.4)	6 (7.4)	30 (7.0)	81 (1.0)

**Table 2:** Association between ethnic categories and prevalence of perceived stress, likely GAD, and likely MDD.

Variable	Ethnic Categories				
	Caucasian *N* (%)	Indigenous *N* (%)	Asian *N* (%)	Other *N* (%)	Total Prevalence *N* (%)
**Perceived Stress**					
Moderate or High Stress ^a^	5593 (85.2)	279 (92.1)	329 (86.1)	618 (88.0)	6819 (85.7)
*Chi square value*	14.71				
*p-value*	<0.01				
Effect Size (Phi)	0.04				
**Generalized Anxiety Disorder (GAD)**					
GAD likely ^b^	2848 (47.1)	144 (51.8)	137 (41.6)	296 (44.7)	3425 (47.1)
*Chi* *square value*	6.4				
*p-value*	0.09				
Effect Size (Phi)	0.03				
**Major Depressive Disorder (MDD)**					
MDD likely ^c^	2695 (43.7)	149 (52.8)	149 (44.2)	305 (47.5)	3298 (44.4)
*Chi Square value*	11.8				
*p-value*	0.01				
Effect Size (Phi)	0.04				

^a^ Moderate or High Stress defined as PSS-10 score ≥ 14; ^b^ Likely GAD defined as GAD-7 score ≥ 10; ^c^ Likely MDD defined as PHQ-9 score ≥ 10.

**Table 3:** Descriptive illustration of mean scores of GAD-7, PHQ-9 and PSS-10 scales by ethnic group.

Variables	*N*		Mean	Std. Deviation	Std. Error	95% Confidence Interval for Mean		Minimum	Maximum
						Lower Bound	Upper Bound		
**GAD-7 Total Score**	**Caucasian**	5750	9.69	5.83	0.08	9.54	9.84	0	21
	**Indigenous**	262	10.56	5.91	0.37	9.84	11.28	0	21
	**Asian**	307	8.65	5.96	0.34	7.98	9.32	0	21
	**Other**	595	9.75	5.99	0.25	9.26	10.23	0	21
	**Total**	6914	9.68	5.86	0.07	9.54	9.82	0	21
**PHQ-9 Total Score**	**Caucasian**	5859	9.30	6.19	0.08	9.15	9.46	0	27
	**Indigenous**	266	11.10	6.50	0.40	10.31	11.88	0	27
	**Asian**	315	9.33	6.82	0.38	8.58	10.09	0	27
	**Other**	612	10.04	6.71	0.27	9.51	10.57	0	27
	**Total**	7052	9.44	6.29	0.08	9.29	9.58	0	27
**PSS-10 Total Score**	**Caucasian**	6243	20.65	6.83	0.09	20.48	20.82	0	40
	**Indigenous**	286	22.56	6.36	0.38	21.82	23.30	8	40
	**Asian**	359	20.55	6.78	0.36	19.84	21.25	2	39
	**Other**	668	21.51	6.91	0.27	20.99	22.04	1	40
	**Total**	7556	20.79	6.83	0.08	20.64	20.94	0	40

**Table 4:** ANOVA comparing sums of squares between and within ethnic groups.

Variables		Sum of Squares	Df	Mean Square	F	Sig.
**GAD-7 Total Score**	**Between Groups**	533.18	3	177.73	5.183	<0.01
	**Within Groups**	236,941.86	6910	34.29		
	**Total**	237,475.03	6913			
**PHQ-9 Total Score**	**Between Groups**	1061.60	3	353.87	8.984	<0.01
	**Within Groups**	277,609.70	7048	39.39		
	**Total**	278,671.30	7051			
**PSS-10** **Total Score**	**Between Groups**	1397.14	3	465.71	10.013	<0.01
	**Within Groups**	351,233.05	7552	46.51		
	**Total**	352,630.20	7555			

**Table 5:** Tukey HSD and Games-Howell post-hoc multiple comparisons.

Dependent Variable	Ethnicity	Ethnicity	Mean Difference	Std. Error	Sig.	95% Confidence Interval	
						Lower Bound	Upper Bound
**^a^ GAD-7 Total Score**	**Caucasian**	Asian	1.04 *	0.34	0.01	0.16	1.92
		Other	−0.06	0.25	0.99	−0.71	0.59
	**Indigenous**	Asian	1.91 *	0.49	<0.01	0.65	3.18
		Caucasian	0.87	0.37	0.09	−0.08	1.82
		Other	0.82	0.43	0.24	−0.30	1.93
	**Asian**	Other	−1.10 *	0.41	0.04	−2.16	−0.04
**^a^ PSS-10** **Total Score**	**Caucasian**	Asian	0.10	0.37	0.99	−0.85	1.05
		Other	−0.87 *	0.28	0.01	−1.58	−0.15
	**Indigenous**	Asian	2.02 *	0.54	<0.01	0.63	3.41
		Caucasian	1.92 *	0.41	<0.01	0.86	2.98
	**Asian**	Other	1.05	0.48	0.13	−0.19	2.29
		Other	−0.97	0.45	0.13	−2.11	0.18
**^b^ PHQ-9 Total Score**	**Caucasian**	Asian	−0.03	0.39	0.99	−1.04	0.99
		Other	−0.74 *	0.28	0.05	−1.46	−0.01
	**Indigenous**	Asian	1.76 *	0.55	0.01	0.34	3.19
	**Indigenous**	Caucasian	1.79 *	0.41	<0.01	0.74	2.84
		Other	1.06	0.48	0.126	−0.18	2.30
	**Asian**	Other	−0.71	0.47	0.44	−1.92	0.51

^a^ Tukey HSD post hoc multiple comparison; ^b^ Games-Howell post hoc multiple comparison; * The mean difference is significant at the 0.05 level.

## Data Availability

The data that support the findings of this study are available from the corresponding author, V.I.O.A., upon reasonable request.
